# Granule cells reorient cortical manifolds to separate contexts but preserve their geometry

**DOI:** 10.64898/2026.03.03.709240

**Published:** 2026-03-04

**Authors:** Martha G. Garcia-Garcia, Michał J. Wójcik, Srijan Thota, Luke Drake, Amma Otchere, Oluwatobi Akinwale, Lizmaylin Ramos, Rui Ponte Costa, Mark J. Wagner

**Affiliations:** 1 National Institute of Neurological Disorders & Stroke, National Institutes of Health, Bethesda, MD 20894, USA.; 2 Department of Physiology, Anatomy and Genetics, University of Oxford, Oxford, UK

## Abstract

To learn effectively, animals must generalize across yet distinguish between related contexts. Generalization relies on low-dimensional neural manifolds found throughout neocortex^1-3^, which accelerate learning by constraining neural activity to task-relevant axes^4,5^. Conversely, context separation is attributed to neural expansion layers that can project information into high-dimensional feature spaces^6-8^, most famously cerebellar granule cells (GrCs)^9-11^. To investigate the generalization-separation tradeoff, we simultaneously imaged key nodes in the universal cortico-cerebellar pathway^12,13^—premotor layer 5 pyramidal tract (L5PT) and GrCs—during parallel learning of two distinct skills with shared temporal structure. Rather than expanding the cortical representations, GrCs retained their low-rank encoding of each task. Across contexts, however, despite stable cortico-cerebellar coupling, L5PT activity patterns generalized while GrC patterns temporally remapped. Mechanistically, GrCs used affine transformations that rotated the cortical manifolds apart but preserved their intrinsic low-dimensional geometry. Moreover, GrCs decorrelated cortical trajectories most strongly in expert animals. This reveals a fundamental architectural division of labor: the cortex generates invariant dynamic primitives for smooth generalization, while the cerebellum reconfigures them to drive context-specific output.

Generalization allows animals to accelerate new learning guided by past experience^14^. In the brain, however, using the vast combinatorial repertoire of theoretically possible neural activity patterns would make generalization and rapid learning computationally prohibitive, creating a “curse of dimensionality.”^1^ To solve this dilemma, the neocortex employs low-dimensional neural manifolds^2,4,5^—population activity patterns constrained to a low-rank subspace in a high-dimensional embedding. By restricting activity to axes capturing essential behavioral variables (e.g., time, speed), manifolds help circuits generalize to previously unseen task variations^15^. They can also facilitate generalization *across* contexts through “structural learning”^16^: using the underlying features of one task to quickly learn another^5^. These advantages strongly incentivize reusing manifolds to make circuits robust^17^ to both within- and cross-context variations. Accordingly, although the cortex can separate related contexts when needed^18^, evidence suggests that whenever possible, the brain prioritizes repurposing low-dimensional neural structures^3,4,19-21^—a fundamental property shared with artificial neural networks^22-25^.

However, manifold reuse comes at a steep cost: interference^6,26^. When two contexts share overlapping neural activity patterns, it is difficult to drive distinct downstream control policies^27^. A classical architecture for distinguishing contexts is the neural “expansion layer”: by projecting overlapping inputs into a high-rank feature space^7,8^, patterns can be maximally separated^9-11^. This motif is ubiquitous in classification circuits: overlapping odor representations are discretized into sparse clusters in piriform cortex^28,29^; smooth representations of visual space transform into categorical objects in inferior temporal cortex^30^; continuous encoding of physical space is converted to a contextual “hash code” in dentate gyrus^31^. Most famously, the greatest capacity for expansion is found in cerebellar granule cells (GrC), which collectively outnumber all other neurons in the brain combined^32^. Yet, while GrCs possess the capacity for high-rank representations^33^, this presents a paradox for manifolds: using high-dimensional expansion to maximally separate points along a low-dimensional structure would “shatter”^34^ the topology required for smooth dynamic prediction and structural learning^6,35-37^.

These opposing constraints create a fundamental generalization-separation tradeoff: low-dimensional manifolds solve the “curse of dimensionality” but invite interference; high-dimensional expansion solves interference but exacerbates the “curse.” A direct interface between these competing demands is the universal cortico-cerebellar pathway that connects the layer 5 pyramidal tract (L5PT) to cerebellar GrCs via the pons^12,13,38^. To investigate how this circuit resolves this dilemma, we simultaneously imaged premotor L5PTs and GrCs during “parallel skill learning,” where animals alternately trained on two tasks with distinct sensorimotor elements but shared temporal structure.

## Results

### Imaging parallel learning of two tasks with shared temporal structure but distinct sensorimotor contexts

**Extended Data Figure 1 ∣:**
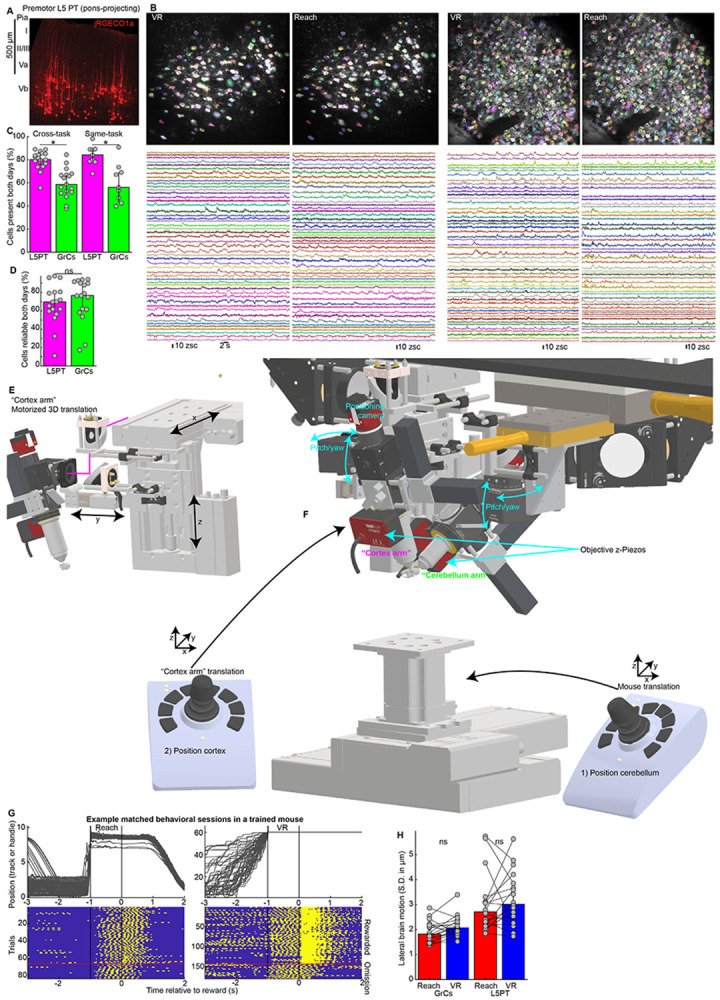
Related to Figure 1 **a,** Histological image of jRGECO1a expression in pons-projecting premotor cortical neurons, which are enriched in the deeper part of Layer V ~700 μm below the surface of the brain. **b,** For the example VR-Reach matched imaging session pair in Figure 1, mean two-photon images show spatial filters for all L5PT and GrC with detected activity in both tasks (138 L5PT and 368 GrCs). Traces show 50 example neurons from each imaging session and cell type with colors corresponding to the cell maps above. **c,** Bars show the fraction of cells detected in each session that also had detected activity in the other session; this fraction was significantly higher for L5PT than for GrCs, likely due to the small size and high packing density of GrCs. However, this difference was indistinguishable in same-task control comparisons, indicating a context-independent noise floor (p=0.0006 and 0.008, respectively). **d,** Bars show fractions of cells in each cross-task session pair that were reliable across both tasks, which did not differ by cell type (p=0.5). **e,f,** Custom dual-site microscope mechanics enabling cross-day registration. To accommodate the challenges of tracking dual-region brain activity while alternating between two distinct behavioral apparatuses, we redesigned a dual-site two-photon microscope^97^ to equip both the “cortex arm” and the mouse platform with joystick-controlled motorized 3D translation. **e,** Isolated view of the redesigned “cortex arm.” Two high-load, long-travel (75 mm), high accuracy Zaber stages provided motorized “x” and “z” control (X-LRQ075HP-DE51). A third compact motorized Zaber stage provided 25 mm of “y” travel (LSA25A). **f,** Overall view of both the motorized cortex arm and the cerebellum arm with 16x 0.8 NA Nikon and 40x 0.8 NA Olympus objectives positioned over the imaging sites on a model mouse skull. Below, a motorized 3D translation platform with 150 mm x/y travel (Zaber X-LRQ150AP-DE51C) and 40 mm z travel (Zaber X-VSR40A) allowed positioning the cerebellum under the cerebellar imaging objective for both behavioral apparatuses. Registration followed a four-step sequence: (1) Align the cerebellar objective optical axis to the window using the “cerebellar arm” pitch/yaw rotational axes. (2) Position the cerebellar imaging site using the motorized translation platform; fine-tune depth via objective z-piezo; (3) Align the cortical objective optical axis using the left arm pitch/yaw rotational axes; (4) Position the cortex imaging site using the motorized cortex arm; fine-tune depth via objective z-piezo. **g,** Single-trial behavioral data from a representative matched Reach-VR session pair. Traces (top) show single position trajectories Rasters (bottom) show binary lick sensor contacts with trials grouped into rewarded and omitted reward blocks for ease of visualization. **h,** Brain motion. Dots show sessions, quantified as standard deviation across all frames of the lateral motion correction computed by the image registration algorithm. Brain motion did not differ between tasks, but was slightly higher for L5PT likely due to the primary skull fixation plate encasing the cerebellar window.

**Figure 1 ∣:**
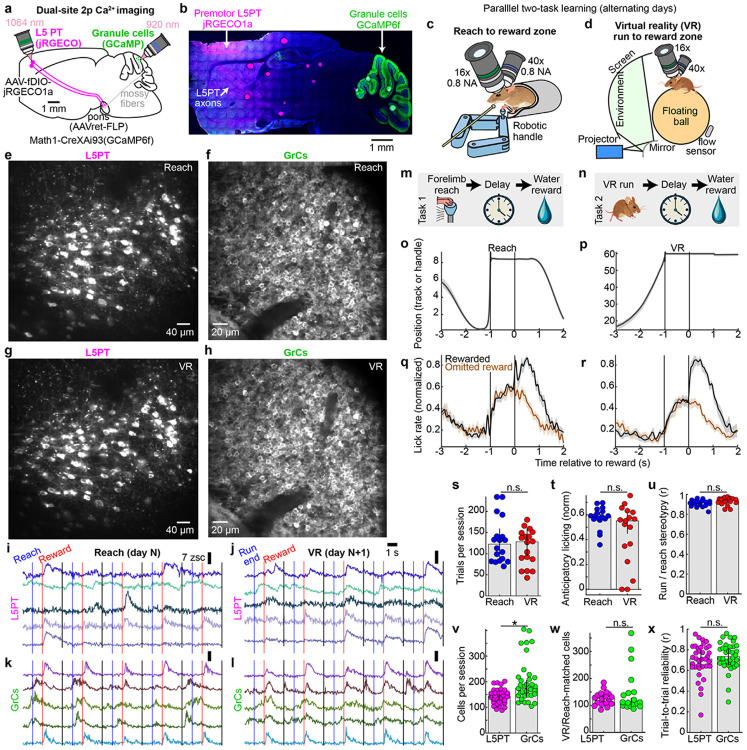
Simultaneous cortex and cerebellar 2p imaging during parallel learning of two novel skills **a,b,** Dual-site imaging strategy. **a**, Schematic of simultaneous two-photon imaging of right premotor cortex pons-projecting layer 5 pyramidal tract neurons (L5PT) and cerebellar granule cells (GrCs) in left Crus I, II and simplex. (**b**) Histology showing L5PTs labeled with jRGECO1a (via AAVretro-FLP in pons and AAV-fDIO-jRGECO1a in premotor cortex) and GrCs labeled with GCaMP6f (via *Math1-Cre;Ai93;ztTA* triple transgenic). **c,d,** Behavioral apparatus for the reach-for-reward task (**c**) and virtual reality (VR) run-for-reward task apparatus (**d**) both imaged with a custom dual-site two-photon microscope (Extended Data Figure 1). **e–h,** Representative fields of view showing the same populations of L5PTs (**e,f**) and GrCs (**g,h**) tracked across Reach and VR sessions. **i-l**, Example simultaneous activity traces from five tracked L5PTs and GrCs during Reach and VR trials. Vertical lines indicate movement offset (blue), reward delivery (red), and trial end (black). **m,n,** Trial structure. Both tasks shared a common timeline: action initiation (Reach vs. Run), delay, reward consumption and inter-trial interval. **o-r**, Behavioral performance. Cohort-averaged position trajectories of the reach handle (**o**) or running track position (**p**, both 18 session-pairs), and corresponding lick rates (**q,r**; normalized per-session, Gaussian smoothed *σ*=33 ms, both 17 session-pairs). These and all subsequent shaded regions denote s.e.m. **s-u**, Task comparability. In reach and VR, animals executed comparable numbers of trials **(s),** exhibited matched levels of anticipatory licking (mean rate [−0.5, 0] s relative to reward; **t**) and movement stereotypy (**u**; all p>0.05, 18 sessions in **s,u**, 17 in **t**). These and all subsequent bar centers and error bars denote median and median absolute deviation of the sample (m.a.d.). **v-x**, Imaging statistics. **v,** Total active cells per session (p=0.0005, 18 sessions each). **w**, Count of cells active, reliable, and tracked across both tasks (p=1, 18 matched VR-reach session pairs). **x**, Response reliability (correlation of odd vs. even trial averages, per-session medians across cells; p=0.3).

High-level task representations are often found in rodent premotor cortex^39,40^, which disynaptically targets GrCs throughout contralateral cerebellar lobules CrusI, CrusII, and simplex^41,42^. We therefore developed simultaneous dual-site imaging of premotor L5PT and GrCs. To image L5PT neurons, we used the red-shifted Ca^2+^ indicator jRGECO1a^43^, excited with a 1064 nm laser for deeper tissue penetration (~700 μm, Extended Data Figure 1a). To specifically target L5PT, we injected AAVretro-FLP into the right pontine nuclei and AAV-fDIO-jRGECO1a into the right premotor cortex (Figure 1a). We concurrently expressed the green Ca^2+^ indicator GCaMP6f in all cerebellar GrCs via the triple transgenic *Math1-Cre;Ai93;ztTA* (Figure 1b). Using a custom dual-site two-photon microscope, we visualized both populations through contralateral cranial windows (Video S1).

To investigate the neural mechanisms of task generalization versus separation, we designed a paradigm where water-restricted mice learned two tasks in parallel: a virtual reality (“VR”) run-for-reward task^44^ and a robotic manipulandum “Reach”-for-reward task^45^ (Figure 1c,d), with comparable training times of ~1-2 weeks. The behavioral apparatuses were engineered to be physically compatible with the microscope geometry, enabling us to revisit the same imaging fields and track neural populations across behavioral contexts (Figure 1e-l, Extended Data Figure 1b-h). In the Reach task, animals stood in a tube and used their left forepaw to push a robotic manipulandum to a threshold distance of 6 mm (maximum extent: 8 mm), after which they waited through a 1-s delay to receive a water reward. Following a 2-s consumption window, the robot automatically returned to the start position. In the VR task, animals stood on an air-suspended ball and self-initiated locomotion through a virtual linear environment to a target zone 60 mm away. Upon reaching the target, the ball locked in place and animals waited through an identical 1-s delay for reward. After reward delivery, the screen darkened during a 2-s consumption and inter-trial interval (ITI) before repopulating at the start location (Video S2).

**Extended Data Figure 2 ∣:**
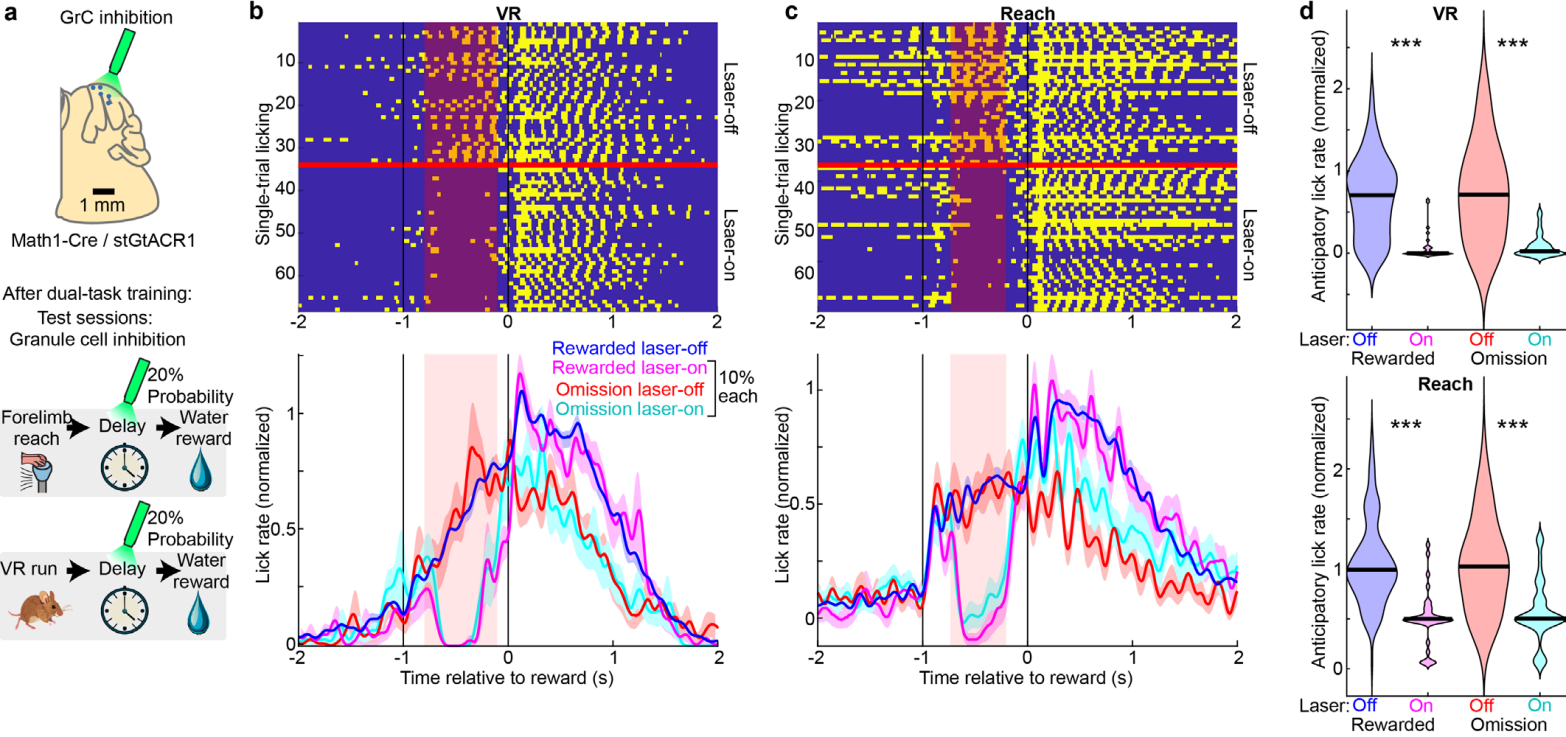
GrC activity is necessary for the expression of anticipatory licking in both tasks, Related to Figures 1 and 2 **a,** Schematic of the optogenetic paradigm for inhibiting GrCs during the delay period on interleaved perturbation trials (4 sessions from 4 mice). 70% of trials were rewarded laser-off; 10% each were: rewarded laser-on; omission laser-off; and omission laser-on. Posterior cerebellum was illuminated through a cranial window with a 594 nm laser in Math1-Cre X stGTACR1 mice. **b,c,** Licking behavior in the VR task **(b)** and Reach task **(c)** during control trials and randomly interleaved GrC inhibition trials (20%). Rasters (top) show binary lick contacts for rewarded laser-off and rewarded laser-on conditions. Traces (bottom) show smoothed lick rates for all four conditions (4 sessions/mice; normalized to the rewarded laser-off condition). **d,** Violins quantify anticipatory licking (mean [−0.7,−0.2] s relative to reward; normalized as above) during all 4 conditions. GrC inhibition trials significantly abolished licking (all laser off vs on comparisons p<7×10^−6^).

The sensorimotor differences between contexts were extensive: tube-constrained versus ball-restrained; dark room versus bright virtual environment; single-limb control versus whole-body locomotion; and manipulation of a handle versus a sphere. Despite these differences, the tasks shared a rigid temporal scaffold: Action→1-s Delay→Reward+ITI (Figure 1m,n). In both contexts, animals learned stereotyped movement trajectories, either of the robotic handle (Figure 1o) or the ball rotation (Figure 1p). Furthermore, animals developed predictive licking time-locked to reward delivery, even on unexpected reward omission trials (Figure 1q,r). In both tasks, animals executed comparable numbers of trials (Figure 1s, Reach: 123±9, VR 128±8), developed comparable anticipatory licking (Figure 1t, Reach: 0.59±0.01, VR: 0.55±0.03), and achieved similar movement stereotypy (Figure 1u, Reach r=0.92±0.004, VR r=0.94±0.004; 18 sessions from 9 mice). Importantly, optogenetic inhibition confirmed that normal GrC activity was required for anticipatory licking in both tasks (Extended Data Figure 2).

We reasoned that different neural circuits might use one of three strategies to encode the two behaviors:

1. Generalization, where overlapping trajectories exploit shared temporal structure for efficient learning^3,4^.
2. Separation through expansion, where overlapping trajectories orthogonalize into a shattered topology^46^.
3. Separation through transformation, where overlapping trajectories reorient apart to drive distinct policies.

Overlapping representations would support transfer of learned timing rules. Crucially, while “shattering” expansion would maximally separate input patterns both within and across contexts, “transformation” would separate the contexts while keeping their internal geometry intact.

### Simultaneously tracking premotor L5PT and cerebellar GrC representations of two-task learning

To compare L5PT and GrC dynamics during the learning of two tasks, we tracked individual neurons across matched "session pairs," consisting of either "cross-task" transitions (VR-Reach) or, as a control, "same-task" transitions (VR-VR or Reach-Reach). Sessions in aligned pairs were typically separated by 1-3 days of training. We applied a strict two-step filter to isolate genuine biological remapping from technical artifacts. First, to avoid conflating biological silence with technical dropout, we restricted our analysis to cells with detected activity on both days of each pair. Retention rates were lower for GrCs than for L5PT (Extended Data Figure 1c, 80±1% L5PT and 59±2% GrC), likely due to the extreme density and small size of GrCs, which complicates cross-day registration. Crucially, however, these retention rates were the same between cross-task session pairs and same-task control session pairs, indicating that tracking efficiency was a behavior-independent noise floor.

Second, to filter out noisy or task-unrelated cells, we calculated a “reliability index” for each cell by correlating its trial-averaged activity on odd versus even trials^47^. We included only cells that were robustly task-locked (*r* >0.4) on *both* days. This criterion demonstrated that most cells participated reliably in both contexts (Extended Data Figure 1d; L5PT: 70±3%; GrCs: 77±4%). By restricting our analysis to “jointly reliable” populations, we established a rigorous baseline: if a cell time locks in both tasks *and* changes its firing pattern, it represents a genuine contextual remap. Overall, this yielded balanced datasets permitting fair comparisons between L5PTs and GrCs. We recorded comparable total cell numbers (Figure 1v, 143±3 L5PT, 175±5 GrCs per session), registered similar numbers of active cells across tasks (Figure 1w, 127±4 L5PT, 112±4 GrCs), and observed equivalent reliability in both populations (Figure 1x, *r_odd:even_*=0.71±0.01 L5PT, 0.74±0.01 GrCs).

### Representations of VR and Reach tasks are broadly similar between L5PT and GrCs

**Figure 2 ∣:**
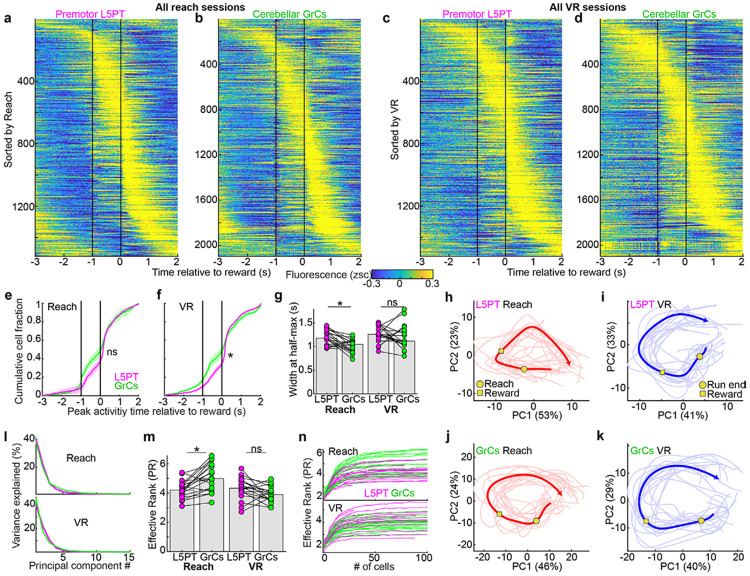
VR and reach tasks recruit common neural populations into similar low-dimensional dynamics. **a-d**, Task-aligned activity rasters, including all neurons active and reliable in both tasks (from 18 matched Reach-VR session pairs in 9 trained mice). Rows show fluorescence averaged across rewarded trials aligned to reward delivery, with reach/run offset at −1 s. Cells are sorted by peak activity time independently for each panel (1,521 L5PT, 2,096 GrCs). **e,f,** Cumulative distributions of peak activity times relative to reward (mean±SEM across sessions). L5PT and GrCs were indistinguishable in Reach (p=0.13) and broadly similar in VR (p=0.02). **g,** Temporal widths of activity peaks were slightly narrower for GrCs than L5PT in Reach but indistinguishable in VR (dots show the median full width at half maximum of trial-averaged activity for each session. Reach, p=0.006, VR, p=0.4). **h-k**, Representative low-dimensional population dynamics. 2D projections of neural activity onto the first 2 PCs (computed independently for each cell type and task in one Reach-VR session pair). Thick lines show the mean; thin lines show 15 single trials most correlated with the mean. Axis percentages display variance explained. **l-n**, Dimensionality analysis. **l**, Scree plots show variance explained by each PC (mean across 18 sessions each, these and all subsequent shaded regions denote S.E.M.). **m**, Effective rank (participation ratio: ${\left(\sum \lambda_{i}\right)}^{2}∕\sum \lambda_{i}^{2})$ for each session. GrC rank was slightly higher in reach (p=0.002) but indistinguishable from L5PT in VR (p=0.1). **n,** Rank saturation (computed on populations subsampled at 20 intervals and averaged across 50 bootstraps). Curves rapidly saturated at cell counts far below total population size, indicating underlying low-dimensional structure.

We first compared L5PT to GrCs in their representations of each task in dual-trained mice. Trial-averaged activity profiles in the VR and Reach tasks appeared grossly similar for both cell types (Figure 2a-d). We quantified this similarity using two metrics. First, the distribution of peak activity times was highly conserved between L5PT and GrCs (Figure 2e,f). In both populations and contexts, ~25% of neurons peaked during the delay period, and ~50% peaked after reward delivery. Furthermore, L5PT and GrCs shared context-specific features, such as the ramping distribution of peak times over the prolonged VR running period, contrasted with a flatter distribution prior to the brief, ballistic Reach. Second, we computed the width at half-max of the trial-averaged activity peaks (Figure 2g,h). Distributions were similar across cell types; small differences in duration (which reached significance in Reach) were likely because jRGECO1a has slower decay kinetics than GCaMP6f (530 ms and 140 ms^43,48^). Thus, L5PT and GrC activity was comparably dense spatially and temporally^49-53^.

We next considered the population structure of neural activity. Due to their high embedding dimensionality (i.e., immense population size), GrCs are postulated to separate input patterns through high-rank expansion^8-11,46^. To test whether GrC activity actually reflects such an expansion of cortical dynamics, we performed trial-averaged principal components analysis (PCA) on each task independently. Visualizing the trajectories of the top 2 principal components (PCs) revealed that both L5PT and GrC activity typically evolved along smooth, cyclic, low-dimensional manifolds in both tasks (Figure 2h-k). To quantify this similarity, we computed the variance explained by each PC. The scree plots were broadly similar for L5PT and GrCs, consistent with comparably low-rank dynamics (Figure 2l). To estimate the effective dimensionality (i.e., rank), we computed the “participation ratio” (PR)^54^. L5PT and GrC activity exhibited similarly low effective rank, with GrCs slightly higher in reach (consistent with their narrower event widths, Figure 2g) and indistinguishable from L5PTs in VR (Figure 2m, GrCs Reach 5.0±0.2, L5 Reach 4.2±0.1, GrCs VR 3.9±0.1, L5PT VR 4.3±0.1). To confirm that this shared low apparent rank was not an artifact of subsampling, we computed PR saturation curves and found that estimates saturated at cell counts well below the total population size (Figure 2n). This conservation of rank is qualitatively inconsistent with a traditional high-dimensional orthogonalizing expansion^35,46,55^.

Critically, low effective rank implies a massive compression of the available sensory and motor state space. In VR, for example, while the animal's behavior involves high-dimensional degrees of freedom—including whole-body coordination for balance and locomotion^56^, whisker kinematics^57^, orofacial movements^58^, and dense optic flow^59^—the majority of neural variance is concentrated across a far smaller set of axes. This suggests that despite the anatomical *opportunity* to expand these high-dimensional sensorimotor features, GrCs instead actively preserved the low-rank cortical structure, effectively rejecting the vast available degrees of freedom. Overall, two tasks learned in parallel with common temporal structure but widely differing sensorimotor contexts elicited population neural representations with grossly similar low-rank structure in both L5PT and GrCs.

### Individual neurons and population modes generalize across contexts in L5PT but remap for GrCs

**Figure 3 ∣:**
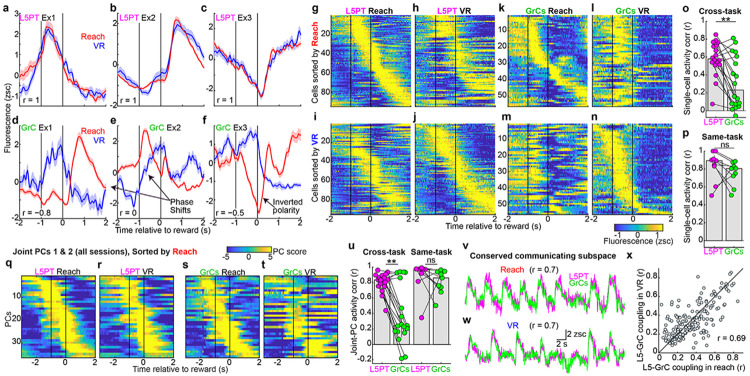
L5PTs generalize across contexts but GrCs temporally remap **a-f,** Representative single neurons tracked across tasks from one session pair. Traces show mean fluorescence across rewarded trials aligned to reward delivery time (186 reach and 120 VR trials). **a-c,** L5PTs exhibiting delay-ramping (**a**), reward-activated (**b**), and delay-suppressed (**c**) dynamics all generalized their temporal profiles across contexts. **d-f,** Simultaneously recorded GrCs exhibited temporal remapping, including phase-shifting from delay-ramping to reward-activated (**d**) or from late- to early-delay activity (**e**), and inverting polarity from ramping to suppressed (**f**). In these and all subsequent analyses, all trial-averaged activity profiles were z-scored to compare timing and minimize cross-session brightness variations. **g-n**, Population activity rasters for all cells active and reliable in both contexts from the session-pair in **a-f** (94 L5PTs and 56 GrCs). Cells are sorted by peak activity time in reach (top row) or VR (bottom row). The L5PT population temporal sequence is grossly preserved across contexts (diagonal structure remains visible in cross-task sorting), whereas GrC temporal sequences largely scramble. **o,p,** Quantification of temporal stability. Pearson correlation of trial-averaged activity profiles for each cell across days (dots show session medians). L5PTs showed higher cross-task similarity than GrCs (p=0.002; 18 session pairs), but were indistinguishable in same-task control comparison (p=0.3; 9 session pairs). **q-t**, Joint-task population dynamics. Rasters show the top 2 joint-task PCs computed on concatenated Reach and VR trial-averaged activity matrices (18 session pairs). Sorting by activity peak time in reach revealed that L5PT population modes generalized across contexts while GrC modes temporally remapped. **u,** Cross-day correlation of the top 2 joint-task PC activity profiles. L5PT population dynamics were substantially more correlated across tasks than GrCs (p=0.002, 18 session pairs), while same-task controls showed no difference (p=0.3, 9 session pairs). Dots show average correlations across PCs 1 and 2 per session pair. **v-x,** L5-GrC communicating subspaces (canonical correlations analysis [CCA] computed across entire VR and reach recording sessions). **v,w,** Representative time-series of the first canonical variable (CV1) across ~20 s of paired VR and reach recordings, demonstrating conserved correlation strength. **x,** Correlation strength of CVs (r>0.1) was highly conserved across Reach vs VR (173 CVs from 18 sessions; p<10^−6^).

We next compared the responses of individual neurons *across* tasks. Surprisingly, individual L5PT neurons exhibited strong cross-context generalization, regardless of their peak response times (Figure 3a-c). In contrast, GrCs displayed robust cross-context temporal remapping. Individual GrCs exhibited complex and heterogeneous forms of remapping, including bidirectional phase shifts and polarity inversions (Figure 3d-f). Consequently, when neurons were sorted by their activity in one task, L5PTs largely maintained their sequential ordering in the opposing task, whereas GrC sequences appeared scrambled (Figure 3g-n). To quantify generalization, we computed the correlation between each cell’s trial-averaged activity in VR and Reach, correcting for signal attenuation^60^ (reliability distributions were equivalent between cell types, Figure 1x). Across recordings, L5PT cross-task activity correlations more than doubled those of GrCs (Figure 3o, r=0.58±0.03 vs 0.23±0.05). Crucially, the cell types were indistinguishable in “same-task” cross-day comparisons (Figure 3p). GrCs were thus more likely to remap activity across contexts, despite remaining active and reliable in both.

**Extended Data Figure 3 ∣:**
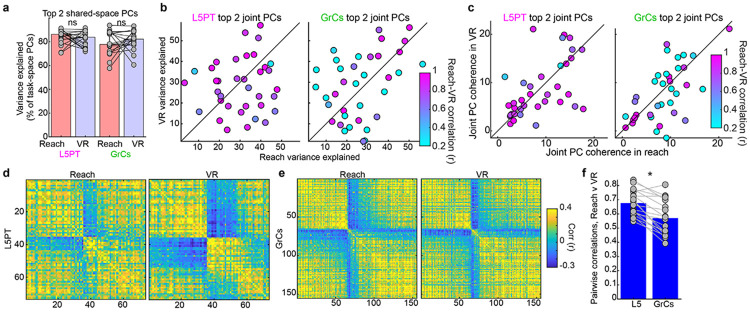
Comparison of GrC and L5PT cross-task dynamics, Related to Figure 3 **a-c,** Goodness of fit for joint-task PCA data shown in Figure 3. **a,** Variance explained by the top 2 joint-task PCs as a percentage of the variance explained by the top 2 “task space” PCs, which was indistinguishable between tasks for both cell types (p>0.1). **b,c,** Joint PC quality variation between tasks. Variance explained **(b)** and coherence **(c)** for all top joint-task PCs (top 2 from all sessions). Coherence is quantified as the ratio of PC variance to the variance of a random projection. Both L5PT and GrCs showed comparable deviation from the unity line, indicating a similar degree of cross-task variation. By contrast, cross-task correlations (color scale) were clearly lower for GrCs—yet this was not associated with a lower PC quality. **d-f,** Pairwise correlations. Consistent with the two tasks being successfully described by a common set of “joint-task PCs,” pairwise neural correlations were moderately preserved across tasks for both cell types. **d,e,** Pairwise single-trial correlation matrices for all L5PTs (**d**) and all GrCs **(e)** from a representative session pair, spectrally sorted to highlight clustering. **f,** Quantification of pairwise correlation preservation across tasks. Both cell types showed moderate preservation, though correlations were more stable for L5PT (p=0.0005; quantified by the Pearson correlation coefficient between the upper triangular portions of the pairwise correlation matrices for the two tasks in each session pair).

We next considered how single-cell differences manifested at the population level. To identify neural ensembles with coherent activations across contexts, we performed ‘joint-task’ dimensionality reduction. We concatenated both tasks’ trial-averaged activity into a single joint matrix and performed PCA^61^. This approach isolates components that capture variance in both tasks, even if their temporal profiles remap. Importantly, these joint components captured the vast majority of variance explained by per-task PCA (L5 ~85%, GrCs ~80%), and they remained coherent in both tasks (Extended Data Figure 3). Visualizing the top joint components revealed a stark contrast: while activity of L5PT modes appeared nearly identical across tasks, GrC modes robustly remapped (Figure 3q-t, PCs 1-2 from all session pairs). Quantitatively, PC cross-task correlations in L5PT more than tripled those for GrCs (Figure 3u, r=0.83±0.01 vs 0.24±0.03; indistinguishable in same-task controls).

This highlighted another fundamental distinction: for L5PT, extracting population modes (‘averaging’ across cells) substantially *increased* cross-task consistency compared to single neurons (rising from *r*=0.58 to 0.83, Figure 3o vs 3u “cross-task”, p=0.0005), implying that cross-context differences were orthogonal to the primary population axes. In sharp contrast, the same population-level analysis failed to rescue cross-context correlations in GrCs (*r*=0.23 vs 0.24, p=0.7). This demonstrates that GrC remapping reflected coherent population-level changes, rather than independent cellular noise. Finally, we tested if GrC remapping stemmed from unstable coupling to the cortex. Using joint-task canonical correlations analysis (CCA), we found that “communicating subspaces” between L5PT and GrCs remained comparably correlated across both tasks (Figure 3v-x). This confirms that, despite a stable, high-fidelity core of cortical coupling, GrCs actively decorrelated task representations that overlapped in the cortex.

### GrCs separate contexts via structured transformations of cortical manifolds

Together, these results raised a paradox: across contexts, individual GrCs seemed to “arbitrarily” scramble their firing times, yet the population-level activity largely occupied the same dominant subspace. To resolve this mystery, we compared the 2D geometry of population trajectories in *independent task spaces* (revealing the intrinsic structure of each manifold) versus the joint-task “*shared space*” (revealing their relative geometric orientation).

**Figure 4 ∣:**
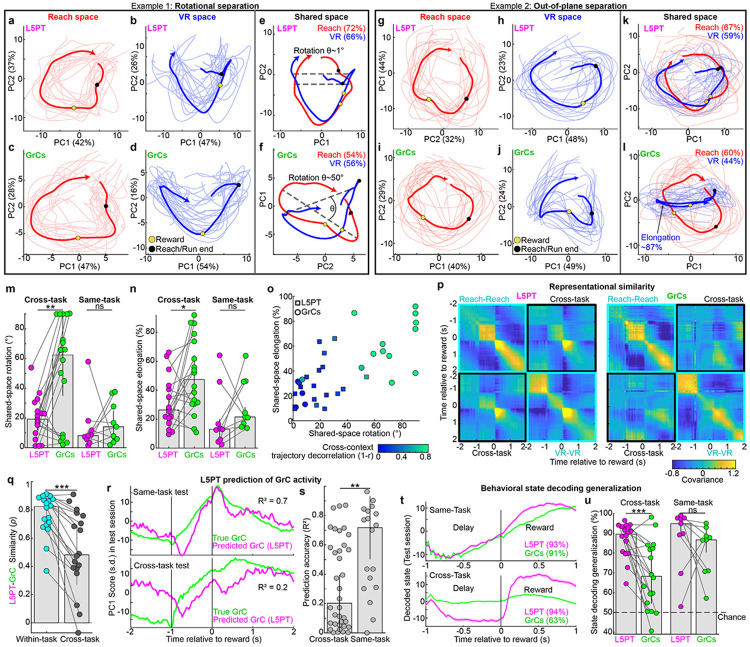
Granule cells separate contexts via geometry-preserving transformations of cortical manifolds **a–l,** Two distinct GrC strategies for separating cortical manifolds. **a–f,** Strategy 1: Rotational separation. Top 2 PCs for L5 and GrCs in independent Reach **(a, c)** and VR **(b, d)** spaces. GrC manifolds preserve fine L5 topological features (e.g., the reach-offset concavity; the triangular VR trajectory shape). **e, f,** Joint Reach-VR PCA ("shared space"). L5 manifolds **(e)** maintain their shape and align, whereas the GrC manifolds **(f)** rotate ~50° apart in shared space, separating the tasks while preserving intrinsic geometry (angle from Procrustes analysis). **g–l,** Strategy 2: "Out-of-plane" separation. **g–j,** L5 and GrC manifolds are similar in the independent task spaces. **k, l,** In shared space, L5 trajectories **(k)** align. However, for GrCs **(l)**, the VR trajectory is “elongated” along PC1—indicating that variance shifted "out of plane" (metric is loss of circularity: $1-\frac{Circ_{shared}}{Circ_{VR}}$). Thick/thin lines denote means and 15 trials most correlated with the mean. Percentages denote variance explained (for shared space, sum of PCs 1+2). Additional examples in Extended Data Fig. 4. **m,n,** Quantification of geometric transformations from the task-specific spaces to the shared space. Across contexts, GrCs exhibited substantially larger relative rotations of the Reach and VR trajectories (**m,** Procrustes angle, p=0.005, 18 session pairs) and greater trajectory elongation **(n,** loss of circularity; p=0.02). By contrast, in control cross-day same-task comparisons, GrCs and L5PTs did not differ by either metric (p>0.3). **o,** Relationship between task-to-shared space geometric transformation and cross-context trajectory decorrelation (markers denote session pairs). Larger rotation and elongation (axes) accompanied greater trajectory decorrelation (color scale; defined as 1-r for PCs 1 and 2). **p,q,** L5 – GrC Representational Similarity Analysis (RSA). **p**, Temporal covariance matrices for L5 and GrCs from a representative reach-VR session pair. Matrices are divided into “within-task” blocks (cyan) versus “cross-task” blocks (black). Within-task, GrC matrices preserved key L5 geometric features (e.g., high activity similarity throughout the delay period in reach and throughout the running period in VR; the sharp transition between the delay and reward consumption periods). Between tasks, however, GrC matrices lacked nearly all features present in L5. Thus, GrCs preserved within-task L5 geometry but broke cross-task L5 geometry. **q,** Quantification (Spearman’s rho) of L5-GrC RSA within versus across tasks (p=0.0002). **r,s,** L5 prediction of GrC activity. A linear decoder was trained to reconstruct the top 2 GrC PCs using the top 20 L5 PCs, then tested on a held-out session from either the same task or the opposing task. **r,** Representative trial-averaged traces of true GrC PC1 activity (green) overlaid with the L5-predicted reconstruction (magenta) for same-task (top) and cross-task (bottom) test sessions. **s,** Prediction accuracy (R^2^). L5PT activity accurately predicted GrC dynamics in same-task tests but poorly in cross-task tests (p=0.008), indicating a context-dependent L5→GrC transformation. **t,u,** Behavioral state decoding (Delay versus Reward). Linear discriminant analysis (LDA) classifiers were trained to discriminate delay from reward using the top 3 shared-space PCs. **t,** Representative trial-averaged LDA projection traces for same-task (top) or cross-task (bottom) test sessions. **u,** Decoding generalization accuracy. State decoding generalized better for L5PTs than GrCs across tasks (p=0.0009) but similarly for same-task tests (p=0.4).

In independent task spaces, GrC trajectories preserved specific topological features present in L5PT. For instance, in “Example 1,” both populations formed a ring with a movement “concavity” in Reach, and a “triangular” cycle in VR (Figure 4a-d). Similarly, in “Example 2” VR, both populations exhibited an outward spiral motif (Figure 4g-j). Thus, within each task, GrCs matched the cortex with smooth, low-rank neural dynamics.

To determine the geometry of the “scrambled” cross-task GrC sequences, we next examined the shared-space projections. L5PT trajectories for VR and Reach remained tightly aligned, confirming manifold reuse (Figure 4e,k). However, the GrC trajectories underwent structured affine transformations, revealing the underlying logic of contextual remapping. In Example 1, the GrC manifolds **rotated** apart in the shared plane, while preserving their intrinsic shapes (Figure 4f). In Example 2, the VR trajectory **elongated** (Figure 4l), indicating that variance rotated “out-of-plane” into orthogonal dimensions. These geometries demonstrated that the cross-context phase shifts and polarity inversions seen in individual GrCs (Figure 3d-f) were in fact single-cell signatures of population-level rotations. Quantitatively, both “in-plane rotation” (Procrustes angle) and “out-of-plane” separation (elongation, i.e., loss of circularity, Methods) were substantially larger for GrCs than L5PT (Figure 4m,n). Crucially, these effects were absent in cross-day same-task controls. Together, the magnitude of these geometric transformations predicted the functional outcome: populations with larger manifold rotations exhibited greater cross-context temporal remapping (Figure 4o). Thus, rather than chaotically remap across contexts, GrCs coherently reoriented entire neural geometries without fragmenting within-task cortical topology.

### GrC transformations faithfully preserve within-task cortical geometry

To quantitatively test whether GrC transformations preserved the underlying cortical geometry, we used Representational Similarity Analysis (RSA)^62^. RSA uses “second-order isomorphism”^63^ to compare the distances between timepoints in neural activity space, and is thus insensitive to differences in the neural ‘coordinate system.’ We computed similarity matrices for both tasks, organizing them into “within-task” versus “cross-task” blocks. Within each task, L5PT and GrC representational geometries were remarkably similar. For instance, in Figure 4p Reach blocks, both cell types displayed a stable population state (high-similarity "square") spanning the delay period [−1, 0] s, with a sharp transition after reward delivery. Similarly, in VR, both populations displayed high similarity throughout the running epoch. Thus, GrCs robustly preserved within-task L5PT geometry (Figure 4q, Spearman’s *ρ*=0.82±0.02).

**Extended Data Figure 4 ∣:**
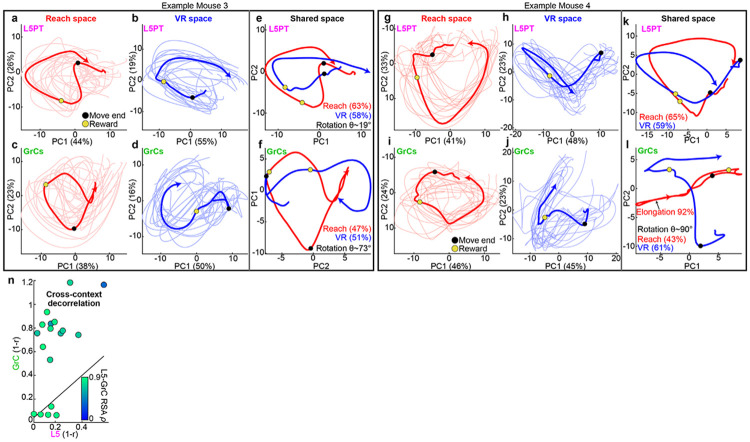
Additional examples of GrC manifold transformations. Related to Figure 4. **a–l,** Low-dimensional trajectory analysis for two additional mice (Mouse 3 and Mouse 4), distinct from those shown in Figure 4. **a–f,** Example Mouse 3: A session characterized by strong GrC rotational separation. **a–d,** Independent task-space projections show smooth cyclic motifs in both cell types and tasks. **e,** In the joint-task shared space, L5PT trajectories remain aligned (manifold reuse, rotation~19°). **f,** GrC trajectories, however, undergo a large relative in-plane rotation (rotation~73°), orthogonalizing the contexts while maintaining the cyclic structure. **g–l,** Example Mouse 4: A session exhibiting concurrent GrC in-plane rotation and out-of-plane separation. **g–j,** Independent projections. **k,** L5PT trajectories remain aligned in shared space (rotation~17°). **l,** GrC trajectories show a complex transformation: the Reach manifold (red) is both strongly rotated (rotation~90°) relative to VR but also significantly elongated (~92%), appearing effectively "collapsed" along PC2. This illustrates that GrC context separation often involves simultaneous in-plane and out-of-plane transformations. **m,** Cross-context decorrelation versus L5-GrC similarity (dots denote session pairs, rho values from Q). Even when GrCs decorrelated contexts far more than L5 (points above unity), L5-GrC similarity remained high (green), demonstrating that GrC orthogonalization of L5 manifolds did not disrupt their local geometry.

Between tasks, however L5PTs and GrCs diverged sharply. In Figure 4p, L5PT displayed similarity *across* tasks, but the GrC cross-task block was dominated by dissimilarity, effectively “blanking out” L5PT’s cross-context generalization (Figure 4q, *ρ*=0.48±0.05). Finally, this cross-task separation did not come at the cost of geometric distortion: we found no correlation between the degree of decorrelation and the fidelity of within-task manifold preservation (Extended Data Fig. 4m). Thus, GrCs solve a dual objective: they actively orthogonalize contexts via structured transformation, but rigorously preserve the cortical topology of each.

### Manifold separation reconfigures the L5-to-GrC transformation and behavioral readout

These results suggested that the GrC transformation of cortical signals is context-dependent. To quantify this, we used L5PT activity (20 shared-space PCs) to predict concurrent GrC activity (2 PCs). We trained a regression model for each session and tested its generalization on a held-out session of either the same task or the opposing task (Figure 4r). Cross-day same-task L5-to-GrC prediction accuracy was high (Figure 4s, R^2^=0.72±0.05), indicating a stable mapping. However, cross-task prediction accuracy collapsed (R^2^=0.2±0.03). Thus, the L5PT-to-GrC transfer function reconfigures across tasks—a consequence of GrC manifold rotation.

This geometric reconfiguration dictates that a fixed downstream “readout” of behavioral state cannot generalize across tasks for GrCs, unlike the stable L5PT scaffold. To explicitly test this, we trained a decoder for each session using the top 3 shared-space PCs to distinguish two behaviorally distinct states conserved across both tasks: the delay ([−1, 0] s) versus the reward period ([0, 1] s). We then tested it on a held-out session from either the same task or the opposing task (Figure 4t). Both L5PT and GrCs decoded behavioral state with high accuracy in cross-day same-task tests (95±1% L5PT, 87±2% GrCs, Figure 4u). In cross-task tests, however, L5PT decoders remained strikingly accurate (90±1%), but GrC decoding accuracy collapsed (68±3%). This confirms that GrCs effectively “encrypt” the cortical temporal scaffold into a context-specific format, preventing a static readout from generalizing across tasks.

### GrC cross-context decorrelation improves with dual-task learning

**Extended Data Figure 5 ∣:**
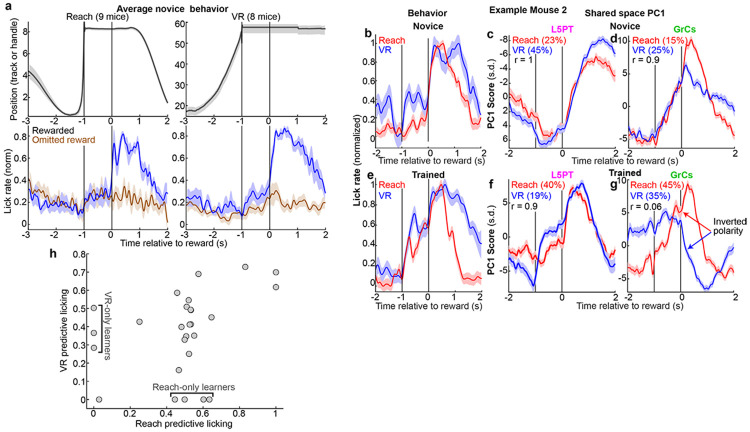
Additional characterization of learning. Related to Figure 5. **a,** Cohort-averaged Novice behavior (analogous to Figure 1) in Reach (left column) and VR (Right column). Top: Position trajectories (reach robotic handle position or running sphere position). Bottom: Lick rates on rewarded and omitted reward trials, showing primarily reactive licking strategies. Traces represent the average of trial-averages from 9 mice (Reach) and 8 mice (VR). **b-g**, A second representative example mouse showing learning-related changes in licking behavior and in both L5PT and GrC joint-task PC1 activity in both Reach and VR (analogous to Figure 5a-f). **h,** Comparison of predictive licking performance in each task independently (quantified per-task as in Figure 5). Brackets highlight 3 sessions with significant predictive licking in VR but not in Reach, and conversely 4 sessions with significant predictive licking in Reach but not in VR, demonstrating that during intermediate learning stages, animals sometimes acquire predictive behavior in only one task, rather than consistently learning both tasks in lockstep.

**Figure 5 ∣:**
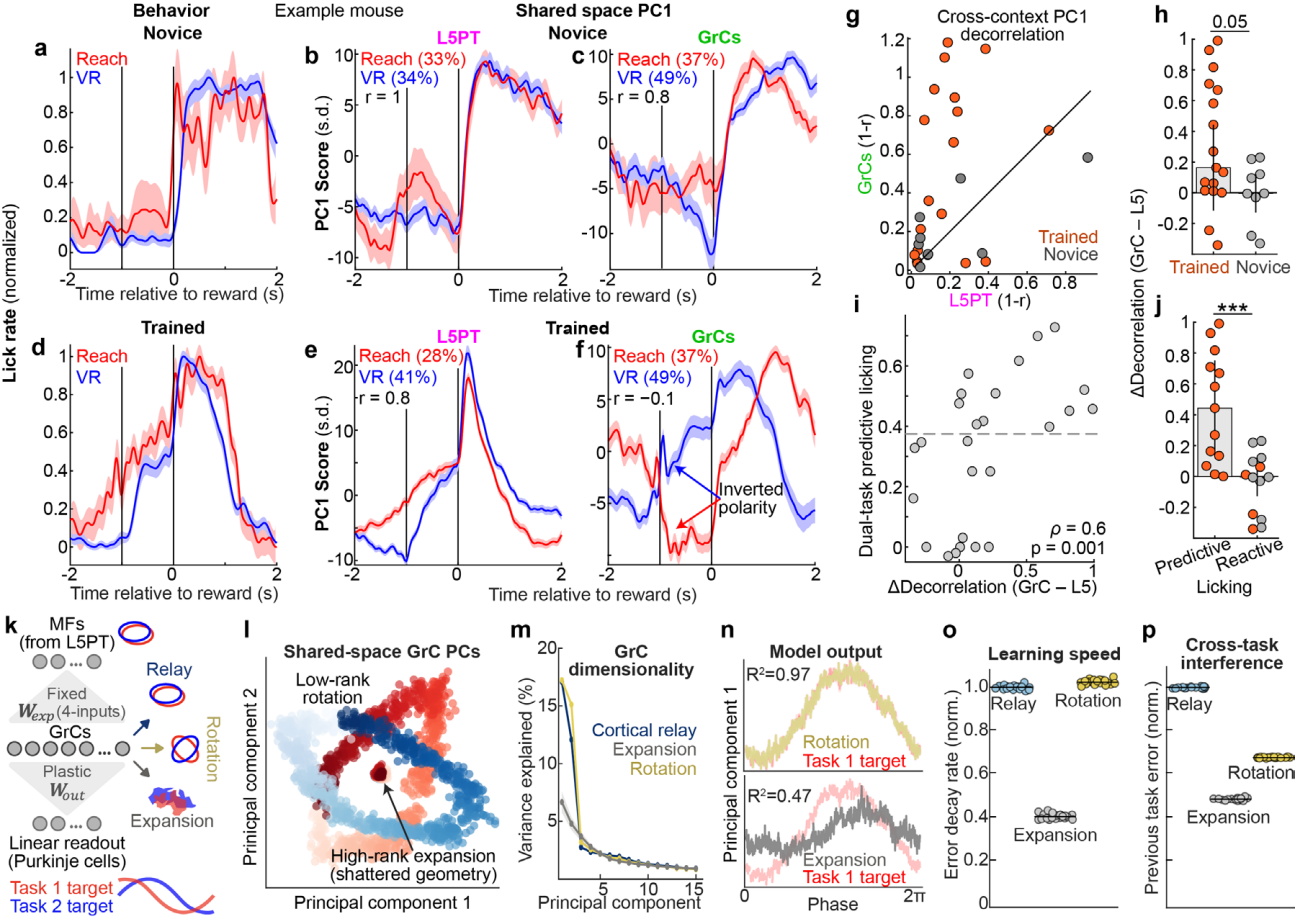
Separating contexts via low-rank transformation facilitates rapid dual-task learning **(a-f)** Emergence of GrC cross-context PC1 decorrelation with dual-task learning. Panels show a representative animal’s Novice session (**a-c**) and Expert session (**d-f**). Neural traces (**b,c,e,f**) show shared-space PC1 time courses; behavioral traces (**a,d**) show normalized lick rates. In Novice, the animal licked reactively after reward delivery (**a**), and both L5 (**b**) and GrC (**c**) PC1 activity strongly correlated between Reach and VR, In Expert, licking converged on a dual-task predictive profile **(d)**, and L5 PC1 retained highly correlated activity across tasks **(e)**. However, GrC PC1 **(f)** exhibited a striking polarity inversion: delay-ramping in VR (like L5) but delay-suppressed in Reach—despite PC1 explaining comparable variance fractions in GrCs as in L5 (percentages). **g,h,** Comparison of L5 and GrC cross-context PC1 decorrelation (1-r). **g,** Scatter plot of Novice and Trained sessions. **h,** Quantification of the ΔDecorrelation (GrC PC1 minus L5 PC1). Trained sessions trended towards more elevated GrC decorrelation relative to L5 (p=0.05). **i,j,** Relationship between GrC-driven decorrelation and behavioral performance. **i,** Scatter plot exhibited positive correlation between ΔDecorrelation (GrC minus L5) and dual-task predictive licking (*ρ*=0.6, p=0.001). Dual-task score was defined as the minimum predictive lick fraction between VR and Reach tasks. For each task, fraction was calculated as $\frac{Lick_{pre}}{Lick_{pre}+Lick_{post}}$, using median rates across trials in [−0.5, 0] s (pre) and [1, 1.5] s (post) windows relative to reward. Dashed line divides the data into the two groups compared in **(j)**. **j,** Grouped comparison. Sessions classified as “Reactive” licking (including both novices and poor-performing trained sessions) exhibited significantly less GrC orthogonalization than “Predictive” sessions (p=0.0009). **k-p,** Simulated dual-task learning. **k**, Schematic of the base network architecture. Continuous kinematic targets (Task 1 and 2) are predicted via a plastic linear readout at the Purkinje cell synapse ($W_{out}$) from fixed GrC representations driven by L5PT mossy fibers. Three distinct GrC architectures were simulated: (1) Cortical relay (shared, overlapping manifolds); (2) High-rank expansion (shattered geometry); and (3) Low-rank rotation (the affine reconfigurations observed in our data). **l,** Latent geometry of GrC activity in each context. Rotation (open loops) orthogonalized the principal manifold axes in state space while preserving their structure. Expansion (center “dot”) collapsed the manifolds into shattered, spectrally whitened representations. **m,** Effective dimensionality. Scree plots of variance explained per PC show that Rotation and Relay models maintained the low-rank structure of the input, while Expansion increased the effective dimensionality. **n,** Simulated output layer trajectories for Task 1 early in training (PC1 at Epoch 10). The Rotation model faithfully tracked the target even early in learning but Expansion failed to reconstruct the temporal scaffold. **o,** Learning speed. Exponential decay rates fit to the training mean-squared error (MSE) loss curves (Extended Data Fig. 6). By preserving low-rank manifold structure, the Relay and Rotation models learned rapidly, while the Expansion model converged slowly due to the increased complexity of the feature space. **p,** Cross-task interference. Performance on the previously trained task during active training of the opposing task (normalized MSE). The Relay model suffered from interference (catastrophic forgetting) due to manifold overlap. The Rotation model mitigated interference comparably to the Expansion model, yet preserved the rapid learning afforded by the low-dimensional geometry. **o** and **p** normalized to the average Relay value.

To investigate the relationship between learning and GrC context separation, we compared novice and trained mice. Most novice animals used a “reactive” licking strategy concentrated after reward delivery in both tasks, and shared-space PC1 activity for both L5PT and GrCs was typically strongly correlated across tasks (Figure 5a-c). In expert animals, behavior converged on a “predictive” strategy with elevated licking before reward, and L5PT PC1 activity remained highly correlated across tasks (Figure 5d,e, Extended Data Figure 5). Conversely, GrC PC1 “inverted polarity” across tasks (Figure 5f). Quantitatively, GrC PC1 cross-task decorrelation significantly exceeded that of L5PT, especially in trained mice (Figure 5g,h). However, “trained” performance varied: for example, some mice acquired predictive licking in only one task (Extended Data Figure 5h). Thus, we next compared the amount of GrC context separation to *dual-task* proficiency (the minimum predictive licking score across both tasks). We found a strong positive relationship: the highest dual-task proficiency often accompanied the strongest GrC context decorrelation (Figure 5i,j *ρ*=0.6). This revealed a striking dissociation: as animals gained dual-task proficiency—resulting in *similar* predictive licking across tasks—their GrC representations became increasingly *distinct*.

### Manifold rotation optimizes the tradeoff between learning speed and interference

We reasoned that neural architectures must navigate a fundamental tradeoff between learning efficiency and context independence. The aligned, low-rank structure of L5PT facilitates rapid learning by constraining the neural state space^5^, but invites interference between contexts. Conversely, the high-rank structure predicted by canonical expansion theory minimizes interference but exacerbates the “curse of dimensionality.” We hypothesized that the coherent manifold transformations we observed in GrCs strike a “Goldilocks”^35^ balance between mitigating interference and accelerating learning using low-rank scaffolds^5^.

**Extended Data Figure 6:**
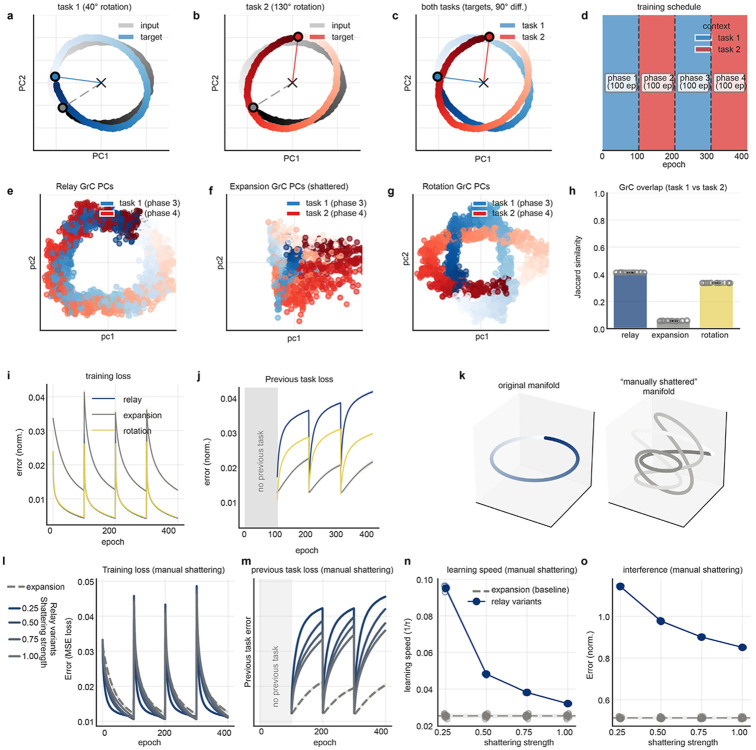
Characterization of simulated architectures and the geometric trade-off between learning speed and interference. **a-c,** State-space geometry of the simulated dynamic prediction tasks, illustrating input manifolds (grey) and temporally shifted target predictions (colored) for Task 1 (40° rotation, **a**), Task 2 (130° rotation, **b**), and both tasks combined (**c**). Noiseless inputs are shown for visualization purposes; all simulations used noisy inputs. **d,** The alternating training schedule, comprising 100-epoch phases. **e-g,** Principal component projections of GrC representations during alternating tasks. **e**, The Cortical Relay model preserves overlapping geometry. **f**, The High-Rank Expansion model reveals a "shattered" high-dimensional geometry (axes scaled independently to each model to visualize structure despite sparsity-induced variance reduction). **g**, The Low-Rank Rotation model geometrically separates the manifolds while preserving low-dimensional topology. **h,** Active GrC population overlap measured by Jaccard similarity. Due to dense coding, both Relay and Rotation models recruit largely overlapping populations. In contrast, the Expansion model sparsifies the representation, resulting in near-zero overlap. This confirms the Rotation model mitigates interference through geometric alignment rather than physical population partitioning. **I,** Training loss over epochs for the three GrC architectures. Note the rapid convergence of the Relay and Rotation models relative to the Expansion model. **j,** Previous task loss (Task 2 loss when trained on Task 1, and vice versa) over epochs. The Relay model suffers from catastrophic interference immediately following task switches. The Rotation model maintains lower error on the inactive task, comparable to the Expansion model. **k-o,** Isolating the geometric effects of manifold shattering. **k**, To test whether geometric fragmentation—beyond the effects of sparsity—drives the learning-interference trade-off, structurally intact (left) or "manually shattered" (right) manifolds were fed into the dense Relay architecture. **l-o**, Increasing the geometric shattering strength within the Relay model progressively degraded training loss (**l**) and learning speed (**n**), while simultaneously reducing previous task loss (**m**, **o**). At maximum shattering, the dense Relay model's performance morphed to match the sparse Expansion model baseline more closely (dashed grey lines).

To test this, we simulated alternating two-task learning in an anatomically constrained mossy fiber-GrC-Purkinje cell circuit with fixed mossy fiber-GrC projections (4 random mossy fiber inputs per GrC; 100-fold expansion) and a plastic linear readout. Input manifolds were overlapping ellipses, mimicking the L5PT data. The tasks required the networks to predict future states along each manifold, but with different time horizons for the two tasks (i.e., different manifold rotational angles; Extended Data Fig. 6a-d). We compared three cerebellar-like architectures with differing GrC representations (Figure 5k-m):

1. “Cortical Relay”: random projection that preserved input geometry.
2. “High-rank Expansion”^8-10,46,64,65^: canonical sparse coding that shatters input geometry.
3. “Low-rank Rotation”: task-specific affine transformations modeled after our data.

The Relay and Rotation models learned the tasks significantly faster than the Expansion model (Figure 5n,o): unlike discrete pattern classification tasks, continuous dynamic prediction tasks require networks to reconstruct *local distances between points* in the input manifolds within each context (mimicking a central Purkinje cell function^66-68^). Consequently, the expansion model learned very slowly due to its “shattered” geometry (and not merely due to sparse coding: learning speed similarly collapsed for dense-coding Relay models driven by artificially fragmented manifolds, Extended Data Fig. 6k-o). *Across* tasks, however, the Relay model suffered from interference (catastrophic forgetting of Task 1 while learning Task 2) due to manifold reuse. In contrast, the Rotation model reduced interference with efficacy comparable to the Expansion model (Figure 5p). Thus, cerebellar manifold rotation solves the generalization-interference dilemma by geometrically separating memories without fragmenting the topology required to rapidly learn dynamic predictions.

## Discussion

By simultaneously recording L5PT cortical output neurons and cerebellar GrC input layer neurons during parallel learning of two new skills, we tested the role of a universal mammalian pathway in resolving the generalization-separation tradeoff. Unlike canonical expansion architectures that shatter topology, we found that GrCs instead faithfully preserved the low-rank neural structures present in the cortex, down to fine geometric details. To separate contexts that shared a cortical manifold without fragmenting its low-dimensional integrity, GrCs performed coherent affine transformations—effectively rotating the manifolds apart. Crucially, GrCs separated the manifolds most strongly in expert dual-task performers. This division of labor solves a fundamental control problem, allowing the brain to efficiently reuse generalized neural dynamics but still drive distinct policies.

The strategy of high-rank expansion—topology “shattering”^34^—is optimal for *discrete pattern classification*^69^ (e.g., odors^28,29^; spatial environments^31^; visual objects^30^). However, cerebellar function is irreducibly *dynamic*, requiring continuous interpolation across time and space^66-68^. For Purkinje cells, which operate as nearly linear integrators^70,71^, continuous interpolation requires preserved Euclidean “distances” in GrC state space^72^. Critically, high-rank manifold expansion violates this geometric constraint: by adding extrinsic curvature^73^, it causes Euclidean and geodesic distances to diverge^74^, which exponentially increases the “covering number” of GrCs required to tile the manifold^75^ (the “curse of dimensionality”). Commensurately, such manifold “crumpling” would catastrophically increase the training samples required to support continuous generalization^76^. Here, we demonstrate that GrCs reject this “maximal separation” strategy. Even the VR task, which required whole-body control during high-dimensional sensory flux, yielded dominant GrC manifolds of only ~rank 4. Instead, by preserving smooth cortical geometries, GrCs likely enable Purkinje cells to generalize via linear interpolation along the manifold. This geometric constraint explains the specific utility of affine manifold transformations for separating contexts: this class of operations uniquely preserves local Euclidean distances^77^.

This raises the question: if rank remains low, why does the brain require an immense number of GrCs? While we investigated the separation of only two neural manifolds, the brain must manage hundreds of contexts—creating a manifold “packing” problem. Because smoother manifolds are inherently larger in neural state space^37^, the massive GrC embedding allows the circuit to separate many cortical manifolds without needing to “crumple” them into smaller volumes. Yet, prioritizing manifold smoothness for continuous interpolation may impose another constraint: many GrCs were active simultaneously. If this “dense” code^49,50^ were “partitioned” by context^9,10,46,64^, it would rapidly saturate the circuit’s capacity. Instead, by maintaining overlapping active populations that reconfigure their *geometry* across contexts, the circuit preserves representational density while mitigating interference. Consequently, while a *single* context exhibits a dense and low-rank code, the *collective* dimensionality of GrC representations can be high—fully utilizing the immense coding capacity.

A critical question concerns the synaptic mechanisms that could support structured transformation. We first considered whether the observed L5-GrC divergence could simply reflect sampling bias rather than a bona fide transformation. Multiple lines of evidence argued against **spatial** sampling bias (i.e., recording from unconnected populations). First, RSA revealed that GrCs faithfully preserved each context’s cortical geometry with high fidelity (*ρ*=0.82, Figure 4). Second, CCA identified stable L5-GrC interaction weights with high predictive power across tasks (Figure 3). Evidence similarly argued against **temporal** sampling bias. While Ca^2+^ indicators act as temporal low-pass filters, our specific indicators actually permitted nearly 4-fold higher temporal resolution for GrCs than for L5PTs. Although this should reveal faster and thus higher-dimensional GrC signals, we found very little difference in effective dimensionality (Figure 2). Instead, these data suggest that L5-GrC reformatting reflects the transformation of shared, low-rank dynamics.

How, then, does the circuit implement structured reformatting? We propose that this geometry emerges when GrCs perform gain-modulated integration of the cyclic cortical dynamics. Since low-dimensional geometries are widespread across the cortex, many GrCs must integrate multiple 'in-manifold' mossy fibers (especially given redundant input sampling^78^). “Remixing” these inputs—effectively rescaling the ‘sine’ versus ‘cosine’ cycle components—directly produces the rotational signatures we observed in individual GrCs (Figure 3d-f). Consequently, if input remixing were structured across the layer as a contextual ‘gain field’^79,80^, the entire population, and thus the entire cortical manifold, would undergo a coordinated transformation. Similarly, enhanced separation with learning (Figure 5) might reflect emergence of a reliable ‘context’ multiplier. Mechanistically, GrCs would therefore operate as a tunable filter bank, where context scales the mixing coefficients that synthesize task-specific affine transformations from a shared set of cortical primitives.

The extensive recurrent connectivity of the neocortex is anatomically suited for generating these stable, low-rank dynamical systems^81^. However, learning such manifolds is computationally expensive, requiring precise synaptic tuning to form a latent landscape^82,83^. This strongly incentivizes reusing dynamical ‘motifs’ across contexts to leverage shared underlying structure^84-86^, unless precluded by incompatible input-output mappings^18,27,87-89^. Yet, even when feasible, reuse entangles distinct neural trajectories^55,90,91^. The cerebellar architecture—defined by extreme numerical expansion—provides a complementary solution. By projecting cortical geometries into a massive embedding space, the cerebellum can separate overlapping trajectories without shattering their low-dimensional geometry. Together these strategies yield an effective division of labor^92^: the cortex specializes in slower learning^93,94^ of generalized dynamic primitives^86,95,96^, while the cerebellum provides the high embedding dimension needed to rapidly reconfigure these for contextually specific policies^38^. More broadly, this structural motif – using expansion layers to coherently reorient low-dimensional manifolds – provides a computational blueprint for continuous prediction and control in biological or engineered systems.

## Materials and Methods

### Mice

All dual-site imaging experiments used Math1-Cre / Ai93 (TIGRE-LSL-TRE-GCaMP6f) / ztTA (R26-CAG-LSL-tTA) mice (aged 6–18 weeks), which express GCaMP6f selectively in cerebellar granule cells (GrCs). Optogenetic studies employed Math1-Cre x stGtACR1 mice. All procedures were approved by the NIH Animal Care and Use Committees.

### Viral Injections

An intersectional viral strategy was used to selectively target pons-projecting L5PT neurons in the premotor cortex. We injected AAVretro-EF1a-Flpo into the basal pons and AAV1-Ef1a-fDIO-jRGECO1a into the premotor cortex. Virus was injected at a titer of ~10^12^ genomes/mL.

Mice were anesthetized with isoflurane (1.5–2% in ~1 L/min O_2_) and mounted in a stereotaxic device (Kopf Instruments). A midline sagittal incision exposed the skull, which was cleared of connective tissue. Two ~300 μm holes were drilled over the pons (0.3 and 0.8 mm right of midline, 3.9 mm posterior to bregma), and 1–2 holes were drilled over the premotor cortex (1.1–1.5 mm right of midline, 0.75–1.75 mm anterior to bregma). Glass capillaries (~25 μm tip diameter) delivered 300–400 nL of AAV-EF1a-Flpo at depths of 5.2 and 5.7 mm below the pial surface at the pontine sites, and 500 nL of AAV-Ef1a-fDIO-jRGECO1a at ~800 μm depth at the cortical sites. Pipettes remained in place for 5 minutes before withdrawal. Expression was allowed to proceed for 4–5 weeks prior to imaging.

### Cranial Window and Headplate Implantation

Cranial windows were implanted 1–2 days after viral injections. To avoid mechanical collision between the cortical and cerebellar objectives, placement of the two windows was planned using computer-aided design (CAD) software.

A ~10 mm x 8 mm patch of skin was excised over the skull. The exposed skull was cleared of soft tissue, and the skin incision edges were sealed with VetBond (3M). A primary cerebellar headplate (1.3 mm thick stainless steel, 5 mm opening) served as the main fixation device, while an auxiliary cortical headplate (1.3 mm thick, 2 screw holes) restricted frontal skull motion. A custom holder ensured consistent relative placement of the two headplates during implantation.

For the cortical window, a 3.5–4 mm craniotomy was centered 1.5 mm anterior to bregma and ~1.5–2 mm right of midline. A 3 mm #0 coverslip glued to a stainless steel ring was positioned at a 20° azimuthal angle from the vertical plane and 20° counterclockwise from the sagittal plane.

For the cerebellar window, the craniotomy was centered over the post-lambda suture and ~2 mm left of midline to access left lobules simplex, crus I, and crus II. The window assembly was depressed onto the brain at a 25° angle clockwise from the sagittal plane and a 50° azimuthal angle from the vertical plane. Windows were cemented with Metabond (Parkell), and headplates were cemented to the skull.

### Virtual Reality (VR) Setup

The custom VR apparatus^44^ consisted of an air-supported 8-inch polystyrene ball (SmoothFoam) and a hemispherical dome displaying a projection reflected via a convex mirror. To facilitate dual-task imaging on the same microscope, the apparatus was modular. The hemispherical dome, projector, and mirror were mounted on a moveable cart. The air-supported ball was mounted on a 12” x 12” breadboard along with head fixation bars, an axle restricting ball rotation to the forward/backward axis, a water reservoir, and solenoids for water delivery and airflow control.

Ball rotation was transformed into VR movement by an optical mouse (Logitech G502 Hero) mounted at the ball’s equator. The VR environment (a 1D track with patterned walls) was generated in MATLAB using ViRMEn software. An Arduino UNO controlled the solenoids and generated a 1 ms pulse per ViRMEn iteration for synchronization with the data acquisition system (PCI-6221, National Instruments, sampled at 5 kHz). The ViRMEn update rate was ~10 ms.

To minimize light contamination, the projection was filtered (Kodak Deep Blue 47 Wratten Filter), and the red-channel PMT (cortex arm) was shielded with Cinefoil (Rosco). Additionally, a custom cover shielded the front lens of the cortical objective (Nikon 16x, 0.8 NA).

### VR Task Structure

Mice were teleported to the start of the track, and the air solenoid opened. After the mouse reached the reward zone (indicated by floor/wall pattern changes), the air solenoid closed, the projection froze, and a water reward was dispensed after a 1 s delay. Trials were aborted if the reward zone was not reached within 30 s. A 2 s inter-trial interval (black screen) followed every trial.

### Forelimb Reach Apparatus

We used a custom two-axis robotic manipulandum following [^45^] with modified data acquisition hardware and motor driver/encoder counting electronics. Device control was programmed in LabVIEW via a CompactRIO chassis (cRIO-9063) communicating with a Windows PC. A 9401 digital I/O module generated PWM signals for the H-bridge motor drivers (Texas Instruments DRV8870EVM). Motor current was read via an ACS70331 sensor and sampled through a 9215 analog input module. DC motor rotation (Maxon DCX22) was measured by an optical encoder (Gurley R120) via a 9411 differential digital input module.

The software utilized nested control loops: a 10 kHz FPGA loop for motor current and encoder readings; a 1 kHz real-time loop for geometric transformations, force calculations, and data buffering; and a high-level Windows PC loop for trial state management and data logging.

### Reach Task Structure

The task required a linear 8 mm reach. Mice self-initiated trials by pushing the handle. A reach >6 mm was scored as successful, triggering a water reward after a 1-s delay. Water was dispensed via a gavage needle equipped with a capacitive lick sensor. Trials terminated if movement ceased (>3 mm distance) for >100 ms. The handle automatically returned to the animal 2 s after trial completion.

### Behavioral Training

Mice were water-restricted (maintained at >80% free-feeding weight) and monitored daily for health markers. Pre-training occurred for 7–10 days prior to imaging.

#### Pre-training:

VR pre-training proceeded in stages: balancing and running short distances (~20 mm) (3–5 days), followed by the full 60 mm track until performance reached ~60 trials in 15 minutes (1–2 days). Subsequently, mice were pre-trained on the Reach task for 1–2 days until reaching comparable pre-training.

#### Training:

After pre-training, animals generally alternated tasks across each successive training day.

### Optogenetic Studies

Math1-Cre mice were crossed with LSL-stGtACR1 mice to generate double transgenics. Following window implantation and one week of single-task training, experts were subjected to perturbation. A ferrule-terminated optical fiber (200 μm core, 0.39 NA) was positioned ~1 mm above the cerebellar window. A 594 nm laser (Coherent OBIS LX) delivered 5–15 mW (CW at fiber tip). During interleaved laser-on trials, a TTL signal triggered the inhibitory opsin in GrCs during the post-movement delay.

### Dual-site two-photon microscope

Building on a previous design^97^, we engineered a custom microscope with two independent, mechanically articulating arms to facilitate chronic dual-task imaging. We incorporated joystick-controlled (Zaber X-JOY3) 3D translation for both the mouse platform and the cortex imaging arm ("left arm"). The mouse positioning assembly utilized two long-travel, high-accuracy linear stages (Zaber X-LRQ150AP-DE51C) for X-Y translation and a high-load vertical stage (Zaber X-VSR40A, 40 mm travel) for Z translation, which supported the weight of both custom behavioral apparatuses via a custom adapter.

The independent cortex imaging arm was mounted on two heavy-duty linear stages (Zaber X-LRQ075HP-DE51, 75 mm travel) for X-Z translation. To accommodate the spatial constraints between the two objectives, Y translation was provided by a compact linear stage (Zaber LSA25A-T4A, 25 mm travel). Custom titanium adapters coupled the translation stages to the imaging assembly, while custom aluminum adapters mated the “floating” elliptic mirrors—required to periscope the beam across the translation stages—to Thorlabs ER rods for x-y-z positioning.

The cortex arm was equipped with a 16x 0.8 NA objective (Nikon CFI75 LWD 16X W) and excited using a 2W 1064 nm laser (Spark Alcor Dual) with a high-speed power modulator (OM6N). The cerebellum arm ("right arm") was equipped with a 40x 0.8 NA objective (Olympus XLUMPlan) and excited using a 2W 920 nm laser (Spark Alcor Dual). Fast volumetric imaging was achieved using objective z-Piezos (Thorlabs PFM450E) on both arms

### Two-Photon Microscopy

Cortex imaging used the red-shifted indicator jRGECO1a excited at 1064 nm, focused to a depth of ~600-800 μm, and with ~70-140 mW of power at the objective. Cerebellar imaging used GCaMP6f excited at 920 nm, focused to a depth of ~100-200 μm, and with ~70-90 mW of power at the objective. Images (512x512 pixels) were acquired at 30 Hz.

Cross-day registration required angular alignment of both objectives to their respective windows using laser back-reflection. Imaging sites were located using motorized translation of the mouse (cerebellum) followed by the cortex arm (cortex). For VR sessions, the cortical site was located prior to placing the custom objective shield, and coordinates relative to visible landmarks (e.g., blood vessel intersections) were recorded to allow precise repositioning. Final alignment involved manual depth tuning via z-Piezo to match the live image to the reference mean two-photon image from the prior session.

### Image Preprocessing

Data were motion-corrected using NoRMCorre (sequential large-displacement rigid followed by small-displacement non-rigid correction). Slow drifts were corrected by dividing out exponential fits to the frame-averaged fluorescence. Source extraction for GrCs and L5PTs was performed using cNMF, followed by manual curation. Traces were obtained by back-applying spatial filters and z-scoring.

### Cross day registration and cell filtering

To track neurons across imaging sessions, we first computed image registration parameters between the mean intensity projection images of each session. These parameters were applied to transform the cell spatial filters from one session into the coordinate space of the other. Cells were initially matched automatically by thresholding the percent spatial overlap between the projected filters and the local filters.

To recover cells that were active in both sessions but missed by the automated sorting in one, we performed a "rescue" step. "Unmatched" filters from one session were projected onto the movie data of the other session. These candidates were then manually curated by verifying the presence of a visible “cell” in the z-scored movie at the times of their fluorescence peak activity timepoints. This procedure was performed bidirectionally (Session 1 onto Session 2, and vice versa). The union of these operations yielded a consensus set of neurons with validated activity in both sessions.

We subsequently filtered this population for response reliability. For each session, we calculated the split-half reliability as the Pearson correlation between the average activity on odd versus even trials, *r_odd:even_*. This raw correlation was corrected using the Spearman-Brown prediction formula: $R_{raw}=2^{*}r∕(1+r)$. To account for differences in trial counts across sessions, we standardized the reliability to a fixed count of 100 trials using the Spearman-Brown prophecy formula: Radj=K∗Rraw∕(1+Rraw(K-1)∗), where $K=100∕N_{\text{trials}}$. Unless otherwise indicated (e.g., Figure 2L-N), all downstream analyses were restricted to neurons that were jointly reliable (*R_adj_*> 0.4) in both sessions of the pair.

### Histology

Mice were perfused with 1x PBS and 10% formalin. Brains were post-fixed, cryoprotected in 30% sucrose, and embedded in OCT. Sagittal sections (50 μm) were stained for GFP (GrCs) and RFP (L5PTs) using chicken anti-GFP and rabbit anti-RFP primary antibodies, followed by Alexa 488 and Alexa 647 secondary antibodies. Sections were imaged on a Zeiss LSM 510 confocal microscope.

## Quantification and Statistical Analysis

Unless otherwise noted, data are reported as median ± median absolute deviation (m.a.d.). Unpaired comparisons used the Mann-Whitney U-test; paired comparisons used the Wilcoxon signed-rank test. Multiple comparisons were corrected using the Bonferroni method.

### Behavioral Analysis

Lick rates were calculated by binning lick sensor events at 1 kHz and smoothing with a Gaussian kernel (sigma = 33 ms). For cross-session comparisons, lick rates were normalized to the peak trial-averaged rate within that session. The **"Predictive Licking Score"** was calculated to quantify the shift from reactive to anticipatory licking. For each task, the score was defined as the ratio of pre-reward licking to total licking: Lick_pre∕(Lick_pre+Lick_post), where Lick_pre is the median rate in the window [−0.5, 0] s relative to reward, and Lick_post is the median rate in [1.0, 1.5] s. Trials were excluded from licking analysis if the capacitive sensor malfunctioned (became “stuck” high across any 1.5 s interval in the trial).

### Single-Neuron Response Metrics

Peak activity times were determined from the maximum of the trial-averaged, z-scored fluorescence trace [−2, 2] s relative to reward. The temporal width of the response was quantified as the Full Width at Half Maximum (FWHM), calculated as the time difference between the half-peak amplitude crossings of the trial-averaged trace. To estimate the true correlation of neural activity across two days ($r_{true}$), we applied Spearman’s attenuation correction to the raw Pearson correlation ($r_{raw}$) of the trial-averaged traces, accounting for the internal reliability ($r_{xx}$ and $r_{yy}$) of the neuron in each context: $r_{true}=r_{raw}\;∕\;\text{sqrt}(r_{xx}*\;r_{yy})$, where $r_{xx}$ and $r_{yy}$ are the split-half reliabilities defined above (odd vs. even trials) for the two sessions.

### Dimensionality Reduction and Effective Rank

Principal Component Analysis (PCA) was performed on the matrix of trial-averaged, z-scored activity in the window [−2,2] s relative to reward delivery (Time x Neurons), after Gaussian smoothing σ=15 ms. The Effective Rank (dimensionality) was estimated using the Participation Ratio (PR): $\text{PR}={\left(\sum \lambda_{i}\right)}^{2}∕\sum \lambda_{i}^{2})$, where lambda are the eigenvalues of the covariance matrix. To assess the robustness of this metric to population size, we computed PR saturation curves by randomly subsampling the population at 20 intervals (from 1 neuron to N) and averaging the PR over 50 bootstrap iterations per interval.

### Joint-Task Dynamics and CCA

To identify neural modes shared across contexts, we performed joint-task PCA. Trial-averaged activity matrices ( [−2,2] s relative to reward) for Reach (X_Reach_) and VR (X_VR_) were independently z-scored and then concatenated along the temporal dimension to form a joint matrix X_Joint_ = [X_Reach_, X_VR_]. PCA was applied to X_Joint_ to yield a common set of eigenvectors (joint PCs). Activity for each task was then projected onto these joint axes.

**Canonical Correlation Analysis (CCA)** was used to identify the "communicating subspace" between L5PT and GrC populations. We applied CCA to the concatenated whole session activity matrices of both tasks to find pairs of canonical variables (CVs) that maximized the correlation between L5PT and GrC activity.

### Geometric Analysis

- **Representational Similarity Analysis (RSA):** We quantified the preservation of manifold geometry using temporal autocovariance matrices. For each task and population, we computed a Time x Time similarity matrix where each element *t_i,j_* represents the dot product of the population activity vectors at timepoints *i* and *j* (i.e., the unnormalized covariance). This metric was chosen to preserve information regarding the magnitude of population activation at each timepoint. To compare L5PT and GrC geometries, we calculated the Spearman rank correlation (*ρ*) between the vectorized upper triangular portions of their respective covariance matrices.
- **Procrustes Analysis:** To quantify the geometric rotation between tasks, we aligned the top 2 PC trajectories of the Reach and VR tasks using Procrustes superimposition (allowing translation, scaling, and rotation). The “Shared-space rotation” was the angular component of this transformation.
- **Circularity (isoperimetric quotient):** To quantify the geometry of neural trajectories, we calculated the Isoperimetric Quotient (often termed Circularity or Form Factor^98^). This was defined as: *Circularity* = 4π*Area/*Perimiter^2^*, where *Area* is the area enclosed by the trajectory (calculated via the MATLAB polyshape function) and *Perimeter* is the total arc length of the trajectory (sum of Euclidean distances between consecutive timepoints). A value of 1.0 indicates a perfect circle, while values approaching 0 indicate increasingly elongated or convoluted trajectories.
- **Elongation (Loss of Circularity):** To quantify the "out-of-plane" warping of trajectories in the shared subspace, we defined Elongation as the reduction in circularity relative to the independent task space: *Elongation* = 1 - (*Circ_shared_* / *Circ_task_*). The summary quantification for each session pair was the maximum of this value across the two paired sessions.

### Decoding and Regression Models

- **L5 ♈ GrC Prediction:** We trained linear regression models to predict the temporal activity of the top 2 GrC shared-space PCs using the top 20 L5PT shared-space PCs as predictors. Models were trained on data from one session and tested on held-out sessions (either "same-task" or "cross-task"). Prediction accuracy was quantified as the squared Pearson correlation (*r^2^* ) between the predicted and actual GrC PC scores, averaged across the top 2 components.
- **Behavioral State Decoding:** We trained Linear Discriminant Analysis (LDA) classifiers to distinguish "Delay" ([−1,0] s) from "Reward" ([0, 1] s) epochs. Input features for training were computed by averaging the activity of the top 2 shared-space PCs across the duration of each epoch for every trial. To visualize the decoding time course (Fig. 4U), we projected the continuous, time-varying activity of the test session onto the discriminant axis identified by the training session. Generalization accuracy (Fig. 4V) was defined as the percentage of correctly classified trial epochs in the test session.

## Neural Network Simulation

### Task geometry

We generated supervised training pairs (x, y) from circular manifolds with intrinsic dimension 2, embedded in a 100-dimensional ambient space. The intrinsic circle was defined as:

$$\tilde{x}(\theta )=r\left[\begin{array}{c} cos\theta \\ sin\theta \end{array}\right],\theta \in [0,2\pi ),r=1$$

To better mirror neural data, we introduced anisotropy using a transform in intrinsic coordinates:

$$S=\left[\begin{array}{cc} 1 & 0.2\\ 0 & 1 \end{array}\right],\;{\tilde{x}}_{base}(\theta )=S\tilde{x}(\theta ).$$

The resulting 2D manifolds were embedded into $R^{100}$ using a random orthonormal basis $E\in R^{100\times 2}$, $E^{T}E=I_{2}$. We then defined two task-specific input manifolds that shared the same base circle, the same shear, and the same embedding plane, differing only by a translation perpendicular to the embedding plane:

$$x_{task1}(\theta )=E{\tilde{x}}_{base}(\theta )$$

$$x_{task2}(\theta )=E{\tilde{x}}_{base}(\theta )+t_{⊥}$$

where $t_{⊥}\in R^{100}$ is constrained to be orthogonal to the embedding plane ($E^{T}t_{⊥}=0$). For each task, we sampled 1000 phases $\theta_{i}∼U[0,2\pi )f$ and added pink noise to obtain the inputs:

$$x_{i}=x_{task_{c}}(\theta )+\sigma \varepsilon_{i}$$

where $\varepsilon_{i}$ is pink noise (β=1), and σ=0.15 and c corresponds to task identity (c∈[1,2]). To construct the targets, we rotated the same base manifold in intrinsic coordinates using task-specific in-plane rotations:

$$R(\phi_{c})=\left[\begin{array}{cc} cos\phi_{c} & -sin\phi_{c}\\ sin\phi_{c} & cos\phi_{c} \end{array}\right],\;\phi_{1}=40^{{}^{\circ}},\;\phi_{2}=130^{{}^{\circ}}$$

The noiseless target manifolds were (Extended Data Fig. 6a-c):

$$y_{task1}(\theta )=\mathrm{ER}(\phi_{1}){\tilde{x}}_{base}(\theta )$$

$$y_{task2}(\theta )=\mathrm{ER}(\phi_{2}){\tilde{x}}_{base}(\theta )+t_{⊥}$$

We then sampled these manifolds with pink noise again to obtain the targets $y_{i}=y_{task_{c}}(\theta_{i})+\sigma \varepsilon_{i}$, with $\varepsilon_{i}$ pink noise (β=1) of σ=0.15. Thus, the dataset consists of paired samples ($x_{i}$, $y_{i}$), where inputs come from unrotated task manifolds and targets from rotated task manifolds, while task separation is preserved by the same perpendicular translation term $t_{⊥}$.

The curriculum consisted of four sequential training phases (100 epochs each): task 1, task 2, task 1, task 2, implementing a continual-learning protocol (Extended Data Fig. 6d). Data were presented in shuffled mini-batches of size *n* = 32 within each phase.

### Model architectures

We compared three cerebellar-inspired neural network architectures designed to investigate how different computational motifs affect continual learning and interference. All models shared a common three-layer structure: an input layer receiving mossy-fiber patterns ($x\in R^{100}$), an intermediate granule-cell layer ($h\in R^{10000}$), and a readout layer producing predictions ($\hat{y}\in R^{100}$). For all models, the readout mapping was identical:

$$\hat{y}=W_{\mathrm{out}}h+b_{\mathrm{out}}$$

Crucially, to mimic the anatomical constraints of the cerebellum, the input connectivity was identical for all three models: the projection matrix $W_{\exp}\in R^{10000\times 100}$ was fixed (untrained) and sparse, where each granule cell received input from exactly 4 randomly selected mossy fibers (4:1 convergence). The models differed only in how the GrC activation h was computed from this sparse input.

1. **Relay model.** The relay model implemented the standard 4-input sparse projection followed by ReLU, but without the normalization or high thresholds required for "shattering." This preserved the manifold geometry in the high-dimensional embedding:
$$h=ReLU(W_{\exp}x)$$
2. **Expansion model (shattering).** The expansion model implemented the canonical Albus/Marr sparse coding theory. It used the same 4-input sparse projection but incorporated L2 input normalization (Golgi cell-mediated feedback^64,65^) and a fixed negative bias to enforce high population sparsity (~1%), which effectively shatters the manifold topology:
$$h=ReLU(W_{\exp}\left(\frac{x}{{\left\|x\right\|}_{2}}\right)+b1),\text{where}\;b=-0.07$$
3. **Rotation model.** This model implemented the interference-reducing geometry observed in our data by applying task-dependent geometric transformations *before* the same 4-input sparse projection:
$$h=ReLU(W_{\exp}{𝒯}_{c}(x)),\text{where}\;c\in [1,2].$$

The input was decomposed into in-plane and out-of-plane components relative to the embedding plane *E*. Transformations (rotation and shear) were applied only to the in-plane component in intrinsic 2D coordinates, then recombined with the preserved out-of-plane component:

$${𝒯}_{1}=\left[\begin{array}{cc} 1 & 0.5\\ 0 & 1 \end{array}\right],{𝒯}_{2}=\left[\begin{array}{cc} cos(90^{{}^{\circ}}) & -sin(90^{{}^{\circ}})\\ sin(90^{{}^{\circ}}) & cos(90^{{}^{\circ}}) \end{array}\right]\left[\begin{array}{cc} 1 & 0.5\\ 0 & 1 \end{array}\right]$$

where task 1 applied only shear and task 2 combined a 90° rotation with shear. The full transformation can be written as:

$${𝒯}_{c}=E{𝒯}_{c}E^{T}\;x+(I-EE^{T})x$$

All linear weights were initialized with Kaiming normal initialization. Sparse masking was applied after initialization to enforce 4-input connectivity per granule cell. Only readout parameters ($W_{\mathrm{out}}$, $b_{\mathrm{out}}$) were trained using Adam with learning rate η=0.0001 and mean-squared error loss:

$$ℒ=\frac{1}{N}\sum_{i=1}^{N}{\left\|y_{i}-{\hat{y}}_{i}\right\|}_{2}^{2}$$

Training was repeated across 20 random seeds for each model type

### Model comparison metrics

#### Variance explained by principal components.

Principal component analysis was performed on granule cell activations (combined across both tasks) to quantify the dimensionality of the population code. For each model architecture and random seed, we computed the proportion of variance explained by the first 20 principal components. Results are reported as mean ± standard deviation across random seeds.

#### Learning speed.

Learning speed was quantified by fitting an exponential decay function to the loss trajectory: L(t)=A⋅exp(−t∕τ)+B, where t is the epoch number, A is the initial decay amplitude, τ is the time constant, and B is the asymptotic loss. Learning speed was defined as 1∕τ, representing the rate of exponential loss decrease (higher values indicate faster learning). To disambiguate learning speed from interference effects, speeds were reported for the first phase only. Fits were performed using nonlinear least squares optimization (scipy.optimize.curve_fit).

#### Previous task loss.

To monitor catastrophic forgetting during continual learning, we tracked the mean squared error on the previous task's data whilst training on the current task. For each training batch on task 2, we computed the loss on task 1 data using the current model parameters without gradient updates (and vice versa).

#### Granule cell overlap (Jaccard similarity).

To quantify the degree of shared neural representations between tasks, we computed the Jaccard similarity of active granule cells. For each sample, we binarized granule cell activations and calculated the Jaccard index as ∣A∩B∣∕∣A∪B∣, where A and B are the sets of active cells for tasks 1 and 2, respectively. We compared activations from models trained to completion on each task (checkpoint after phase 1 for task 1; checkpoint after phase 2 for task 2), then averaged Jaccard scores across all samples and compared random seeds across model types.

### Input geometry shattering control experiment

To test whether the geometric complexity of the input manifold accounts for differences in learning speed and cross-task interference, we conducted a geometry shattering experiment. A sinusoidal transform was applied to the input manifold prior to all network layers, progressively distorting its smooth circular structure in the ambient space while preserving the norm of each point. The transform consists of I folds, where each fold is a single sinusoidal displacement in a random ambient-space direction that oscillates around the ring. Summing I folds with independent directions, frequencies, and phase offsets produces a complex, high-frequency “crumpling” of the manifold geometry. Specifically, for each fold i, a random unit vector $d_{i}\in R^{100}$ was drawn from the full ambient space, an integer-valued random frequency $f_{i}∼U(2,I)$ and phase offset $\varphi_{i}∼U(0,2\pi )$ were sampled, and the displacement

$$x^{\prime }=x+a\sum_{i=1}^{l}sin(f_{i}\theta +\varphi_{i})d_{i}$$

was applied, where a is the displacement amplitude and θ is the angular position of each point projected onto the manifold's embedding plane. The result was then rescaled to the original norm. Increasing I increases the geometric complexity of the manifold as seen by the readout layer, while leaving the task structure and all network parameters unchanged (for visualisation, I was normalised to a shattering strength s∈[0,1] across the tested range.This transform was applied to the relay model’s input across a range of shattering levels, and compared against the expansion model baseline (unshattered). If input geometry were the driver of slow learning and increased interference in the expansion model, shattering the relay input should push its behavior progressively toward the expansion model.

## Extended Data

## Supplementary Material

Supplement 1 Video S1 ∣ Dual-site cortico-cerebellar imaging. The left panel shows L5PT neurons (red, jRGECO1a) in the premotor cortex, and the right panel shows granule cells (green, GCaMP6f) in the cerebellum recorded simultaneously during behavior (2x speed).

Supplement 2 Video S2 ∣ Dual-task performance. The left panel shows an expert VR session and the right panel shows an expert Reach session recorded on the following day in the same animal, with reward times aligned in both tasks and indicated by the appearance of red squares (2x speed).

## Figures and Tables

**Table:** Key Resources Table

REAGENT or RESOURCE	SOURCE	IDENTIFIER
**Antibodies**		
Rabbit anti-RFP	Abcam	62341
Chicken anti-GFP	Aves Labs	GFP-1010
Alexa 488 Goat anti-Rabbit	Abcam	AB150077
Alexa 647 Goat anti-Chicken	Abcam	AB150171
**Virus**		
AAV-Ef1a-Flpo	Addgene	55637
AAV-Ef1a-fDIO-jRGECO1a	Addgene	128317
**Mice**		
Mouse: Math1-Cre	Jackson Labs	Stock# 011104
Mouse: Ai93 (TITL-GCaMP6f)-D	Jackson Labs	Stock# 024103
Mouse: ztTA	Jackson Labs	Stock# 012266
Mouse: LSL-stGtACR1	Jackson Labs	Stock# 037380
**Software and Algorithms**		
MATLAB	Mathworks	https://www.mathworks.com
NoRMCorre	Simons Foundation	https://github.com/flatironinstitute/NoRMCorre
ScanImage	MBF Biosciences	https://www.mbfbioscience.com
LabVIEW	National Instruments	http://www.ni.com
cNMF	Simons Foundation	https://github.com/dylkot/cNMF
ViRMEn	Princeton	https://pniweb.cpaneldev.princeton.edu
